# An Optical System of Star Sensors with Accuracy Performance Varying with the Field of View

**DOI:** 10.3390/s23218663

**Published:** 2023-10-24

**Authors:** Xiaoheng Wang, Xinrou Chen, Zhikun Li, Jun Zhu

**Affiliations:** State Key Laboratory of Precision Measurement Technology and Instruments, Department of Precision Instrument, Tsinghua University, Beĳing 100084, China; wangxiaoheng0013@163.com (X.W.); chenxr98@mail.tsinghua.edu.cn (X.C.); zhkunli@mail.tsinghua.edu.cn (Z.L.)

**Keywords:** star sensors, field focal length, single-star measurement accuracy, field of view

## Abstract

The field of view and single-star measurement accuracy are crucial metrics for assessing the performance of a star sensor. The field of view determines the spatial range of stars that can be captured by the sensor, while the single-star measurement accuracy determines the precision of attitude determination and control for the star sensor. The optical system of conventional star sensors is constrained by imaging relationships. Once the detector is determined, improving either the field of view or the single-star measurement accuracy will result in the degradation of the other. To address this issue, we propose an optical system for star sensors with accuracy performance varying with the field of view. By controlling the relationship between the field focal length of the optical system and the field of view, it is possible to simultaneously enhance both the field of view and the single-star measurement accuracy. We have designed corresponding optical systems to address the requirements for improving the single-star measurement accuracy and field of view. The design results confirm the feasibility of this star sensor. The star sensors are capable of simultaneously meeting the requirements for star pattern recognition and attitude determination, presenting broad application prospects in fields such as space navigation.

## 1. Introduction

Star sensors are sensors used for high-precision attitude measurement of spacecraft, playing a critical role in spacecraft attitude control and navigation tasks [1,2,3]. By utilizing stars in the sky as reference points, the star sensor determines the spacecraft’s attitude by measuring the positions of stars, thereby providing accurate positioning and navigation information [4,5].

From the perspective of working modes, star sensors mainly have two modes: sky area recognition and star tracking. Sky area recognition refers to star map matching in a large sky area, which is the initial working stage of the star sensor. In this phase, the star sensor captures star maps and performs star point extraction, star map recognition, and attitude determination. Star map recognition refers to the comparison of the captured star map with a prestored navigation star catalog, obtaining the right ascension and declination information corresponding to the stars [6]. Upon successful recognition, the star sensor enters the phase of stable stars tracking. In the tracking phase, there is no need for star map recognition, only a need to observe the target star in the very small field of view (FOV), extract the target star points, and perform attitude determination. In tracking mode, the star sensor can output attitude information continuously and stably, and the effective FOV is less than one percent of the entire FOV [7].

A star sensor’s primary technical indicators include the update rate, dynamic range, FOV, single-star measurement accuracy, etc [8,9,10]. These indicators are interrelated. The update rate refers to the refresh rate of the star sensor’s output attitude, mainly determined by the exposure time of the star sensor and the speed of data processing. In space missions, spacecraft may experience changes in lighting conditions, such as intense light or shadows. Dynamic range refers to the range within which the star sensor can adapt to and process different lighting conditions. FOV and single-star measurement accuracy are two indicators that affect the accuracy of star map recognition and attitude determination. The FOV of a star sensor determines the extent of the celestial sky it can capture. A larger FOV allows for the detection of a greater number of stars, thereby improving the speed and accuracy of star pattern recognition [11]. The single-star measurement accuracy determines the precision of spacecraft attitude determination. Higher single-star measurement accuracy leads to more accurate spacecraft attitude determination.

In order to address various space missions and environmental conditions, it is necessary to research methods for enhancing the performance of the star sensor based on its operating environment and mode of operation. Reference [12] describes a transmissive star sensor with imaging quality close to the diffraction limit. The stray light of the star sensor can be well suppressed by designing a light shield. In Reference [13], a transmissive star sensor with a large relative aperture and a wide FOV is proposed, this system exhibits excellent thermal deformation characteristics. Reference [14] proposes a short-wave infrared star sensor applied to near-Earth space navigation, which has both long focal length and large aperture. For conventional star sensors, the focal length of the optical system and the detector jointly determine the FOV and single-star measurement accuracy. Once the detector is determined, increasing the optical system’s focal length enhances the single-star measurement accuracy but reduces the FOV. Conversely, decreasing the focal length can expand the FOV but lowers the single-star measurement accuracy.

The constraint relationship between single-star measurement accuracy and FOV limits the detection capability of star sensors. To address this issue, we propose a star sensor based on the principle of the field focal length (FFL). This star sensor’s optical system has a focal length that varies with the FOV, making the measurement accuracy a function of the field angle. By designing a well-suited FFL, the star sensor can simultaneously enhance the FOV and the single-star measurement accuracy, thus improving its capabilities for star pattern recognition and attitude determination.

We analyzed the imaging principles of the FFL-type star sensor. In response to the requirements for expanding the FOV and improving single-star measurement accuracy, we designed transmissive and catadioptric optical systems. For the transmissive system, the single-star measurement accuracy at the center field exceeds 0.35 mrad, with an FOV of 18°. Under this measurement accuracy, the conventional optical system with the same detector can only achieve an FOV of 10.8°. For the catadioptric system, the single-star measurement accuracy is better than 0.015 mrad, with an FOV of 1.8°. If maintaining the same 1.8° FOV, the single-star measurement accuracy of a conventional optical system is only 0.02 mrad. The design results confirm the advantages of the star sensor in expanding the FOV and improving the measurement accuracy. Compared to traditional star sensors, the system contributes to enhancing the capabilities of star pattern recognition and attitude determination, offering significant application value in spacecraft control and navigation fields.

## 2. Principles

**Figure 1:**
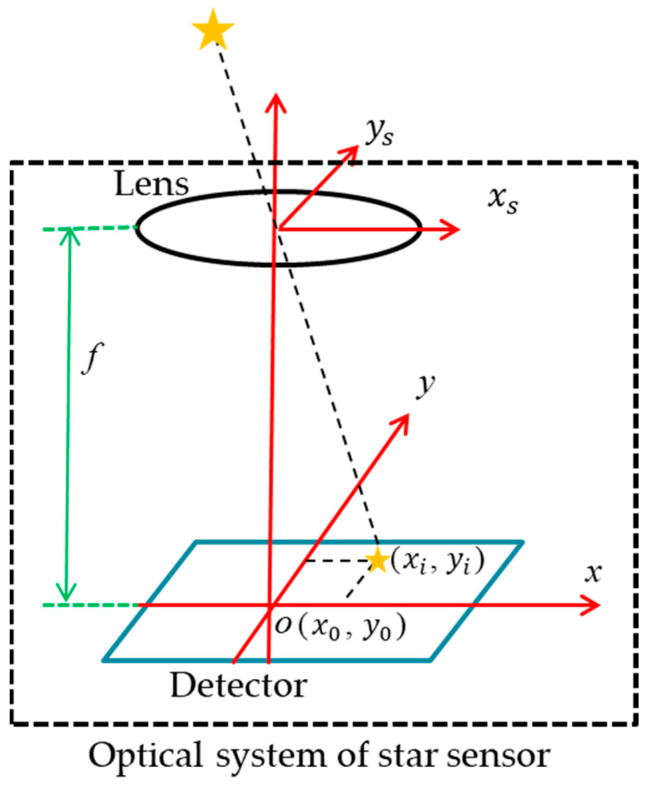
Imaging model of star sensors.

The imaging principle of the star sensor is shown in Figure 1. The optical system focuses the light emitted by stars on the detector. The focal length of the optical system is *f*, the origin of the detector is located at point O(*x*_0_,*y*_0_), and the imaging position of the star point is (*x_i_*,*y_i_*). The position vector *w_i_* of the star’s image point in the star sensor’s coordinate system is denoted as [15,16]

(1) $$w_{i}=\frac{1}{\sqrt{{\left(x_{i}-x_{0}\right)}^{2}+{\left(y_{i}-y_{0}\right)}^{2}+f^{2}}}\left[\begin{array}{c} -(x_{i}-x_{0})\\ -(y_{i}-y_{0})\\ f \end{array}\right]$$

The position of a star in the celestial sphere coordinate system can be expressed as the right ascension *α* and declination *δ* [17,18]. According to the conversion relationship between the spherical coordinate system and the rectangular coordinate system, the direction vector of a star in the rectangular coordinate system is given using the following equation [19]:

(2) $$v=\left[\begin{array}{c} \cos\alpha \cos\delta \\ \sin\alpha \cos\delta \\ \sin\delta \end{array}\right]$$

Under the ideal condition, the relationship between the position vector *w_i_* and the direction vector *v* can be expressed as follows [15]:

(3) $$w_{i}=Av$$

where *A* is the attitude matrix of the star sensor. When there are two or more navigation stars available, the attitude matrix of the star sensor can be solved [20].

**Figure 2:**
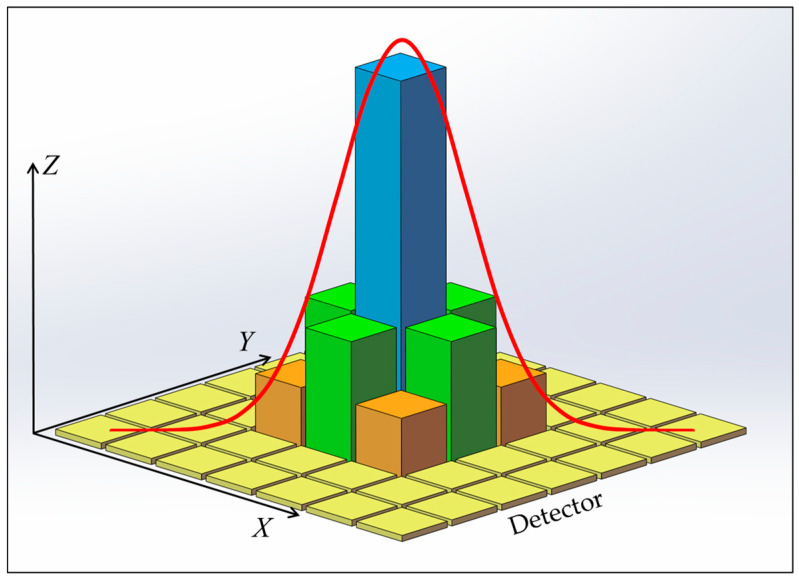
Schematic model of the detector’s output signal.

In the actual imaging process, starlight does not focus into a perfect point, and the energy converging on the focal plane of each FOV is close to the Gaussian distribution [21,22]. The signals output on the detector are illustrated in Figure 2, where the *X* and *Y* axes represent the pixel array of the detector, and the *Z* axis represents the signal values. Due to the influence of diffraction, aberration, and detector sampling, the exact positioning of the imaging location to a point (*x_i_*,*y_i_*) is not possible. Sub-pixel centroiding techniques can reduce the deviation of the star’s imaging position, but they are still unable to reduce the deviation to zero [23]. According to the imaging model shown in Equation (1), the larger the focal length *f*, the smaller the impact of the deviation of the imaging position (*x_i_*,*y_i_*) on the position vector *w_i_*, resulting in higher precision in spacecraft attitude determination.

**Figure 3:**
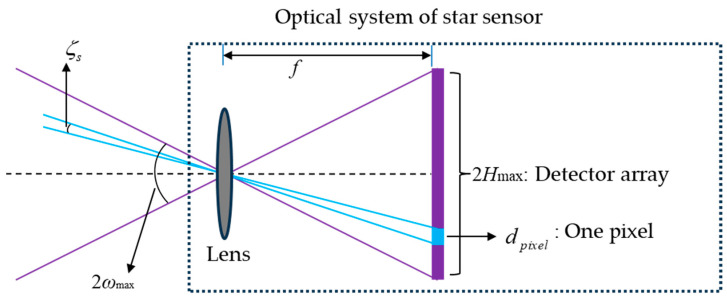
FOV and single-star measurement accuracy of star sensor when centroiding precision is one pixel.

As shown in Figure 3, for conventional optical system of star sensors, the relationship between the half angle *ω*_max_ of FOV and the focal length *f* is as follows [24]:

(4) $$\tan\omega_{\max}=\frac{H_{\max}}{2f}$$

where *H*_max_ is the image height corresponding to the maximum field angle, which depends on the size of the sensitive surface on the detector. The conventional optical system of star sensors has the same focal length in the full FOV, which makes the single-star measurement accuracy $\zeta_{s}$ close to a constant value across the full FOV, which is [25]

(5) $$\zeta_{s}=\frac{2\omega_{\max}}{N_{pixel}}\delta_{c}$$

where *δ_c_* is the centroiding precision of the imaging point, and *N_pixel_* is the number of pixels corresponding to the maximum image height. According to Equations (4) and (5), after the detector is selected, the enlargement of the FOV will lead to a reduction in the single-star measurement accuracy. On the other hand, increasing the focal length can improve the single-star measurement accuracy, but it will reduce the effective detection FOV.

In order to simultaneously meet the requirements for star pattern recognition and attitude determination, we aim for the star sensor to possess both a wide FOV and high single-star measurement accuracy. Different from conventional optical systems, a special optical system with an FFL varying with the FOV exists, which results in different resolutions at different field angles [26,27]. The FFL is defined as the derivative of the image height H to the target field angle ω [28].

(6) $$FFL(\omega )=\frac{dH}{d\omega }$$

For rotationally symmetric optical systems, the size of the detector is

(7) $$H(\omega_{\max})=2\int_{0}^{\omega_{\max}}FFL(\omega )d\omega$$

When d*H* represents the pixel size *d_pixel_* of the detector, the single-star measurement accuracy of the FFL-type star sensor can be expressed as

(8) $$\zeta_{s}(\omega )=\frac{d_{pixel}}{FFL(\omega )}\delta_{c}$$

Equations (7) and (8) indicate that both the image height and single-star measurement accuracy are functions of *FFL*(*ω*). By designing an appropriate *FFL*(*ω*), it is possible to achieve the effects of expanding the FOV and improving the single-star measurement accuracy, thus meeting the requirements for both star pattern recognition and attitude determination.

## 3. System Design

### 3.1. Expanding FOV

Expanding the FOV is beneficial for the star sensor to capture more stars, thereby improving the speed and accuracy of star pattern recognition. The star sensor is equipped with a detector with a single pixel size of 5 μm, with 540 pixels arranged along the imaging dimension. We require that the single-star measurement accuracy of the star sensor reaches 0.35 mrad under the condition that the centroiding precision *δ_c_* is 1 pixel. Using Equations (4) and (5), it can be deduced that the total FOV of the conventional optical system of star sensors is approximately 10.8°, with a focal length of 14.3 mm. We aim to expand the total detection FOV based on the imaging principle of FFL without compromising the single-star measurement accuracy. Therefore, it is necessary to determine *FFL*(*ω*) according to the demand for FOV expansion and design the corresponding optical system.

For rotationally symmetric optical systems, the entire FOV can be characterized by a “line field” perpendicular to the optical axis. In this case, the *FFL*(*ω*) can be described by the polynomial shown in Equation (9).

(9) $$FFL\left(\omega \right)=\sum_{i=0}^{n}k_{i}\omega^{i}$$

where *k_i_* is the coefficient and *ω* is the radian value corresponding to the field angle. The optical system’s center FOV is set to have the optimal measurement accuracy, where the single-star measurement precision meets the design requirement of 0.35 mrad. Moreover, the star sensor also needs to satisfy the image height constraint condition given by Equation (7). The single-star measurement precision at the center FOV and the image height of total FOV form two constraints, which means that selecting the first two terms from the *FFL*(*ω*) polynomial is sufficient to meet the design requirements. In this case, *FFL*(*ω*) can be expressed as

(10) $$FFL\left(\omega \right)=k_{1}\omega +k_{0}$$

The single-star measurement accuracy at the center FOV should be better than 0.35 mrad. According to Equation (8), the *FFL*(0) > 14.3 mm. That means the FFL value at the system’s center FOV should be consistent with the focal length of the conventional system. To allow for a certain margin of precision, the FFL at the center FOV is set as

(11) FFL(0)=15

Expanding the FOV of the star sensor from 10.8° to 18°, and substituting the radian of the half FOV and the detector size into Equation (7), yields

(12) $$H_{\max}=2\int_{0}^{\frac{\pi }{20}}FFL(\omega )d\omega$$

By solving Equations (10)~(12), the result is

(13) FFL(ω)=−81.56ω+15

**Figure 4:**
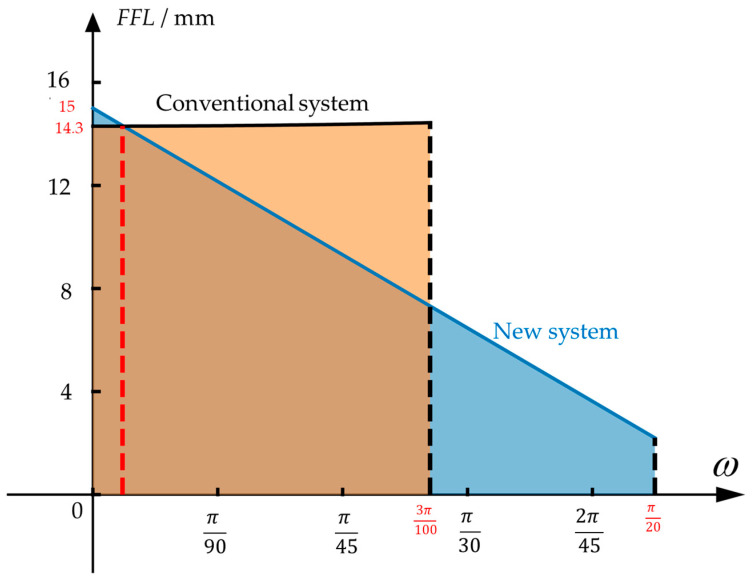
The *FFL*(*ω*) of the conventional system and the new FFL system of star sensor. In the figure, the angle where the red dotted line is located is the field angle when the conventional system and the FFL system have the same focal length. The yellow and blue shadow areas are the image heights of the conventional system and the FFL system, respectively.

The comparison between the *FFL*(*ω*) as shown in Equation (13) and the conventional system is illustrated in Figure 4, where the vertical and horizontal axes represent the FFL value and the field angle, respectively. The field angle of the conventional system is 10.8°, and the radian corresponding to the half field angle is $\frac{3}{100}\pi$. After expanding the FOV by optimizing *FFL*(*ω*), the field angle can reach 18°, and the radian corresponding to the half field angle is $\frac{\pi }{20}$.The shaded area under the *FFL*(*ω*) curve represents the image height. Since both systems use the same detector, the shaded areas under the two curves are the same. The *FFL*(*ω*) function shown in Equation (13) exhibits the characteristic of decreasing with the increasing field angle, which allows the new star sensor to have a larger observation field.

**Figure 5:**
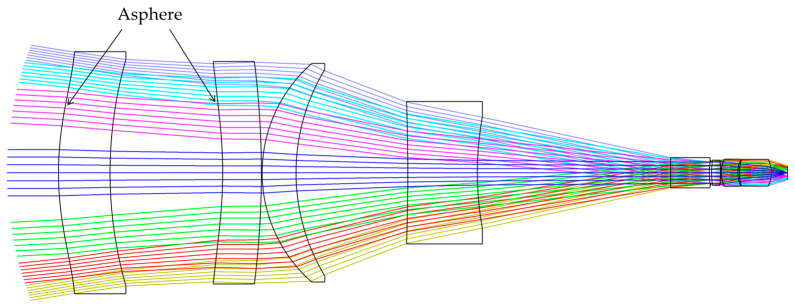
The optical system of the FFL-type star sensor. The different colored lines in the figure represent light beams coming from different angles of view.

Based on the *FFL*(*ω*) derived from Equation (13), a merit function should be developed to design the corresponding optical system. In addition to aberrations, the merit function should also include constraints for image height and resolution for each FOV, as shown in Equations (7) and (8). In order to improve the image quality, the front surfaces of the first and second lenses of the optical system are optimized as aspheres. Figure 5 shows the designed optical system, which consists of eight lenses.

**Figure 6:**
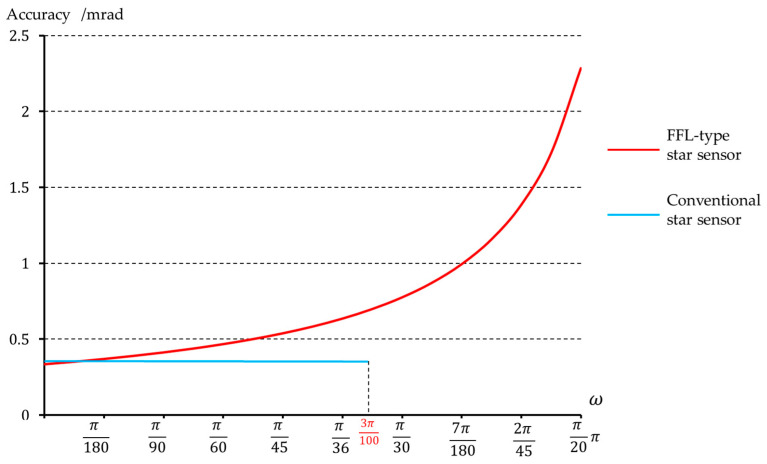
Single-star measurement accuracy of the FFL-type and conventional star sensor.

Table 1 shows a comparison of the relevant optical parameters between this system and the conventional system. Under the condition of consistent image height at the maximum FOV, the FFL-type star sensor can increase the FOV from 10.8° to 18°. Figure 6 illustrates the single-star measurement accuracy of the star sensor after expanding the FOV, achieving a precision of 0.33 mrad at the center field, which meets the accuracy requirements for attitude measurement.

The FFL-type star sensor also has advantages in improving measurement accuracy. If the detector is not replaced and the FOV of the conventional star sensor is expanded to 18° to match the FOV of our designed star sensor, then according to Equation (4), the focal length of the optical system for the conventional star sensor would be only 8.5 mm. Using Equation (5), the single-star measurement accuracy of the traditional star sensor is calculated to be 0.58 mrad, while for our designed FFL-type star sensor, the single-star measurement accuracy is 0.33 mrad. The FFL-type star sensor can enhance the measurement accuracy of attitude to 1.76 times that of the conventional system.

**Figure 7:**
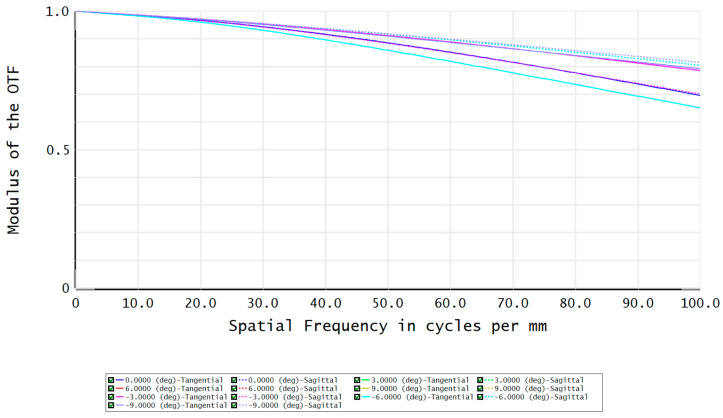
The MTF of the optical system.

**Figure 8:**
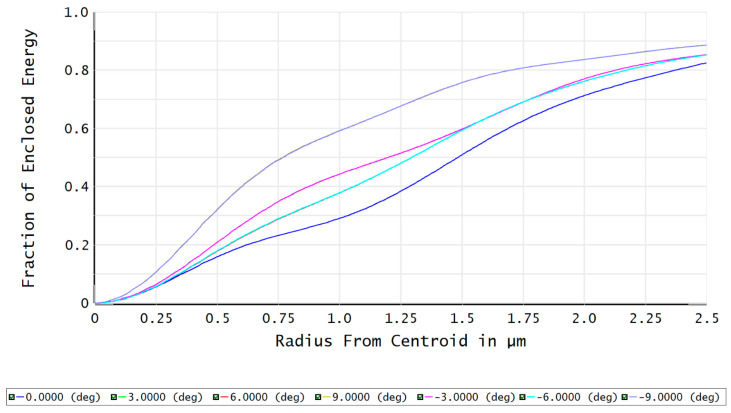
The fraction of enclosed energy of the optical system.

The modulation transfer function (MTF) of the FFL-type star sensor’s optical system is shown in Figure 7. At a Nyquist frequency of 100 lp/mm, the MTF reaches above 0.65, indicating good imaging contrast. The fraction of enclosed energy represents the ability of the star sensor to gather starlight, where a higher energy concentration on a single pixel facilitates the centroid localization of star points. Figure 8 shows that more than 82% of the energy in each field is concentrated within a circle with a 2.5 μm radius, which is advantageous for the subsequent sub-pixel centroid localization.

### 3.2. Improving the Single-Star Measurement Accuracy

The catadioptric optical system is characterized by a large aperture and long focal length, making it a preferred choice for high-precision star sensors [29]. For a conventional Ritchey–Chrétien (R-C) optical system with an aperture of 170 mm and a focal length of 592 mm, using a detector with a pixel size of 12 μm and an imaging dimension with 1550 pixels, the FOV for this system is 1.8°. According to Equation (5), the single-star measurement accuracy of a conventional R-C system is 0.02 mrad when the centroiding precision is 1 pixel.

We aim to further improve the single-star measurement accuracy of the R-C system using the FFL imaging principle and increase the measurement accuracy to 0.015 mrad. According to Equation (8),

(14) FFL(0)=800

With the same detector, the optical system still has to maintain a detection space of 1.8°, then

(15) $$H_{\max}=2\int_{0}^{\frac{\pi }{200}}FFL(\omega )d\omega$$

By combining Equations (14) and (15), we can obtain

(16) FFL(ω)=−26484ω+800

**Figure 9:**
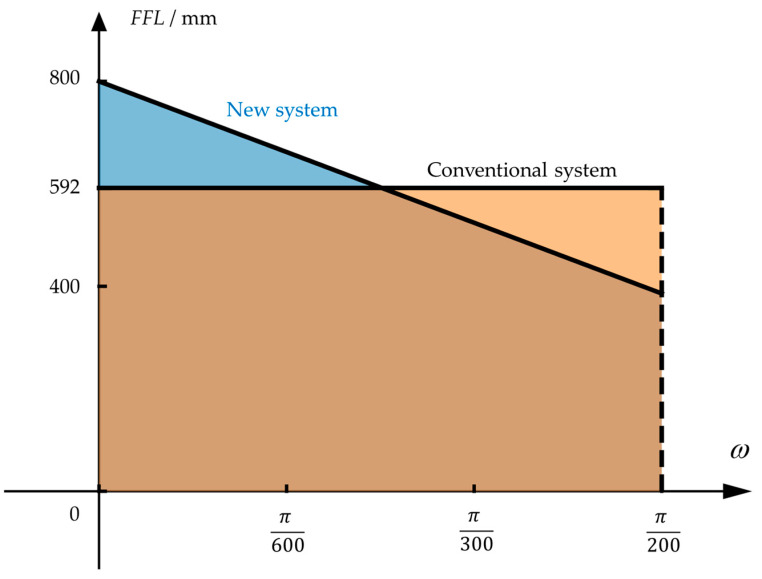
The FFL(ω) of the conventional and new R-C systems.

The comparison between the *FFL*(*ω*) as shown in Equation (16) and the conventional R-C system is shown in Figure 9. Both systems have a half FOV of 0.9°, which corresponds to a radian of $\frac{\pi }{200}$.The FFL-type system has a focal length of 800 mm at the center field, which is 1.35 times that of the conventional system. The enhancement in focal length will improve the single-star measurement accuracy.

**Figure 10:**
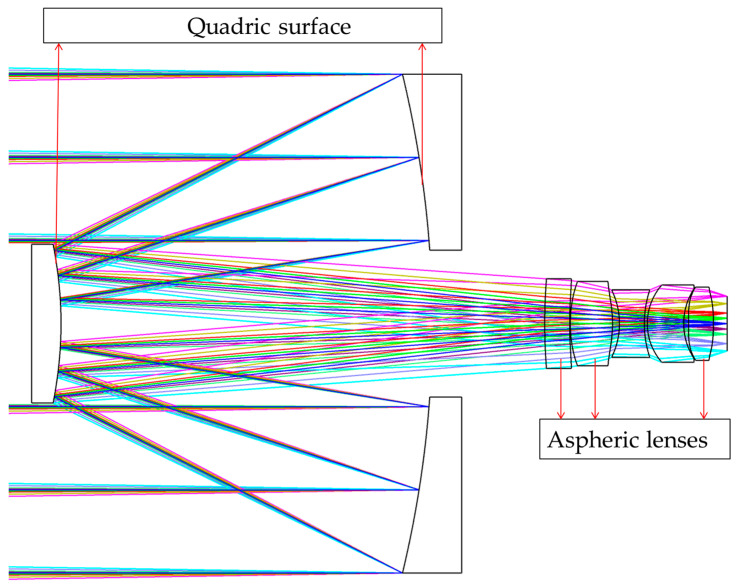
The FFL-type R-C optical system of the star sensor. The different colored lines in the figure represent light beams coming from different angles of view.

Based on the conventional R-C system, we incorporate the constraints of the *FFL*(*ω*) on image height and resolution to perform the actual design of the system. It is essential to note that in order to reduce manufacturing costs and complexity, we should utilize transmission corrector lens groups to primarily control *FFL*(*ω*), while the two reflection mirrors mainly achieve the implementation of long focal length at the center field. Figure 10 shows the FFL-type R-C system, where both reflection mirrors are quadratic, and the first two and last corrective lenses are double-sided aspherical.

**Figure 11:**
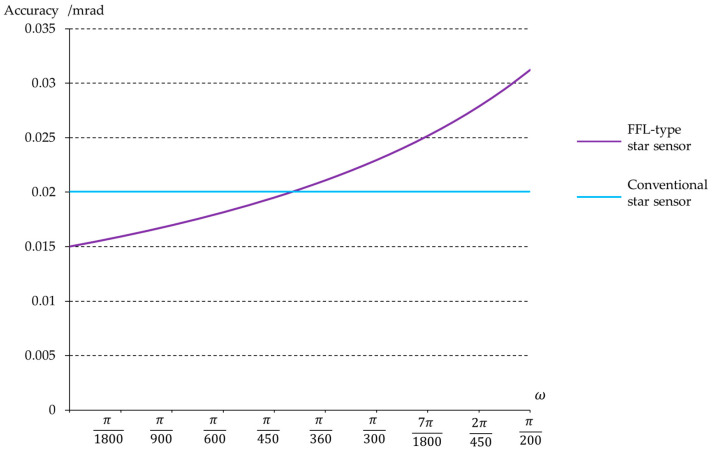
Single-star measurement accuracy of the FFL-type R-C system.

Table 2 shows the relevant parameters for the conventional R-C system and the FFL-type R-C system. The FFL-type R-C system maintains the same detection space while increasing the focal length of the center field. Figure 11 shows the single-star measurement accuracy of the FFL-type R-C system, achieving a precision of 0.015 mrad in the center field. This improvement compared to the conventional system can enhance the spacecraft’s attitude determination accuracy.

**Figure 12:**
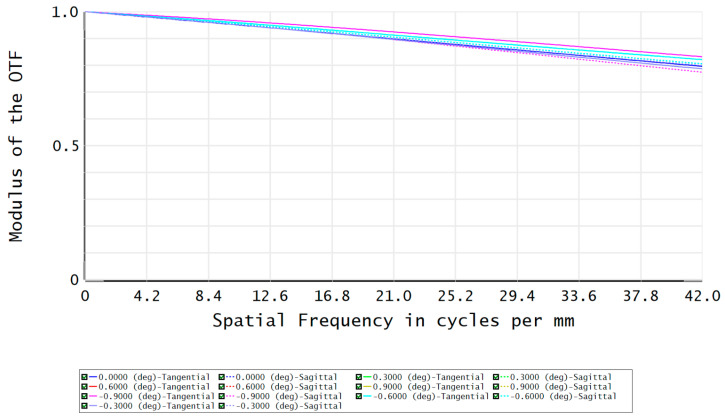
The MTF of the FFL-type R-C system.

**Figure 13:**
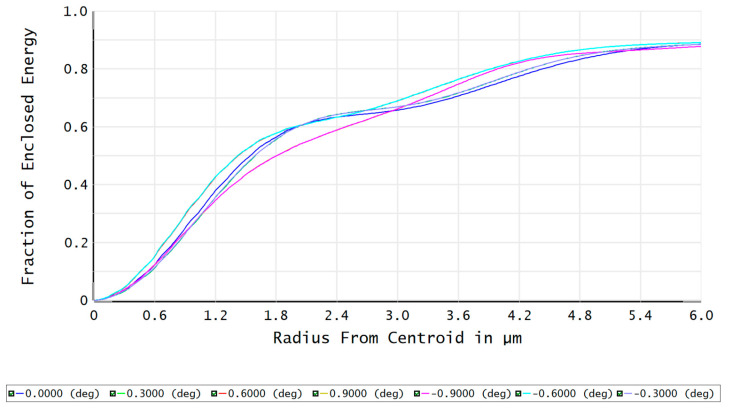
The fraction of enclosed energy of the FFL-type R-C system.

The MTF and fraction of enclosed energy of the FFL-type R-C system are shown in Figure 12 and Figure 13, respectively. The MTF at the Nyquist frequency is greater than 0.75, and more than 87% of the energy in each field angle can be converged within a single pixel. This indicates that the optical system has excellent imaging quality, demonstrating the feasibility of improving the single-star measurement accuracy based on the FFL imaging principle.

### 3.3. Uncertainty Analysis

Due to the need to expand the FOV and improve the single-star measurement accuracy of star sensors, we optimized the *FFL*(*ω*) to design two optical systems, both of which exhibit accuracy performance that vary with the FOV. It is impossible for the machining and assembly of the optical components of star sensors to be completely consistent with the design values. Tolerance factors such as the curvature, thickness, and material of optical elements will have a certain impact on the accuracy performance of the star sensors. Therefore, it is necessary to conduct tolerance analysis based on the actual processing capabilities of the optical system. The tolerances of the system are shown in Table 3, where *λ* is the wavelength of the interferometer, with a value of 632.8 nm.

**Figure 14:**
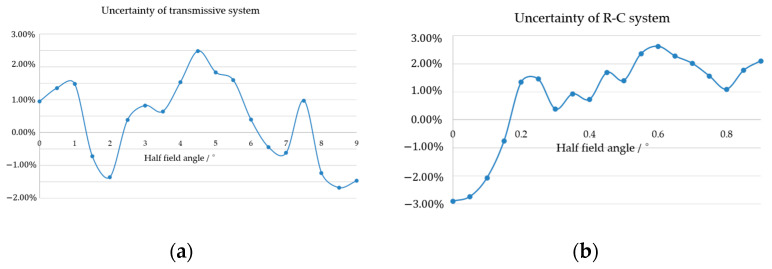
The uncertainties of the two FFL-type star sensors: (**a**) transmissive system; (**b**) R-C system.

Analyses of the tolerance performance of the single-star measurement accuracy for the two FFL systems are shown in Figure 5 and Figure 10. The uncertainty of the system’s single-star measurement accuracy is defined as

(17) $$A_{dev}=\frac{E(\zeta_{sr})-\zeta_{s}}{\zeta_{s}}\times 100\%$$

where $E(\zeta_{sr})$ is the average value of single-star measurement accuracy after Monte Carlo tolerance analysis, and $\zeta_{s}$ is the design value. Figure 14a,b depict the uncertainty of the FFL-type transmissive and R-C star sensors, respectively. It can be seen that the uncertainty of single-star measurement accuracy is controlled within 3% under the achievable processing technology, which indicates the practicability of the system.

## 4. Discussions

Star sensors require different FOV ranges for star pattern recognition and attitude determination. Star pattern recognition requires a large FOV to capture as many stars as possible. On the other hand, attitude determination only needs to track known stars, so the required FOV is less than 1% of the total, but the precision in star measurements is highly demanding. Conventional star sensors face a trade-off between the single-star measurement accuracy and the FOV range after the detector is determined. Improving either of these indicators will lead to a decrease in the other. The FFL-type star sensor that we proposed can independently control the measurement accuracy for different field angles. By designing a reasonable *FFL*(*ω*), it can simultaneously expand the detection FOV and improve the single-star measurement accuracy.

By utilizing the FFL’s imaging principle, it is also possible to reduce the size of the detector while maintaining the FOV and single-star measurement accuracy. Reducing the number of detector arrays can lower the system’s cost, decrease the data readout time, and improve the star sensor’s update rate [30].

**Figure 15:**
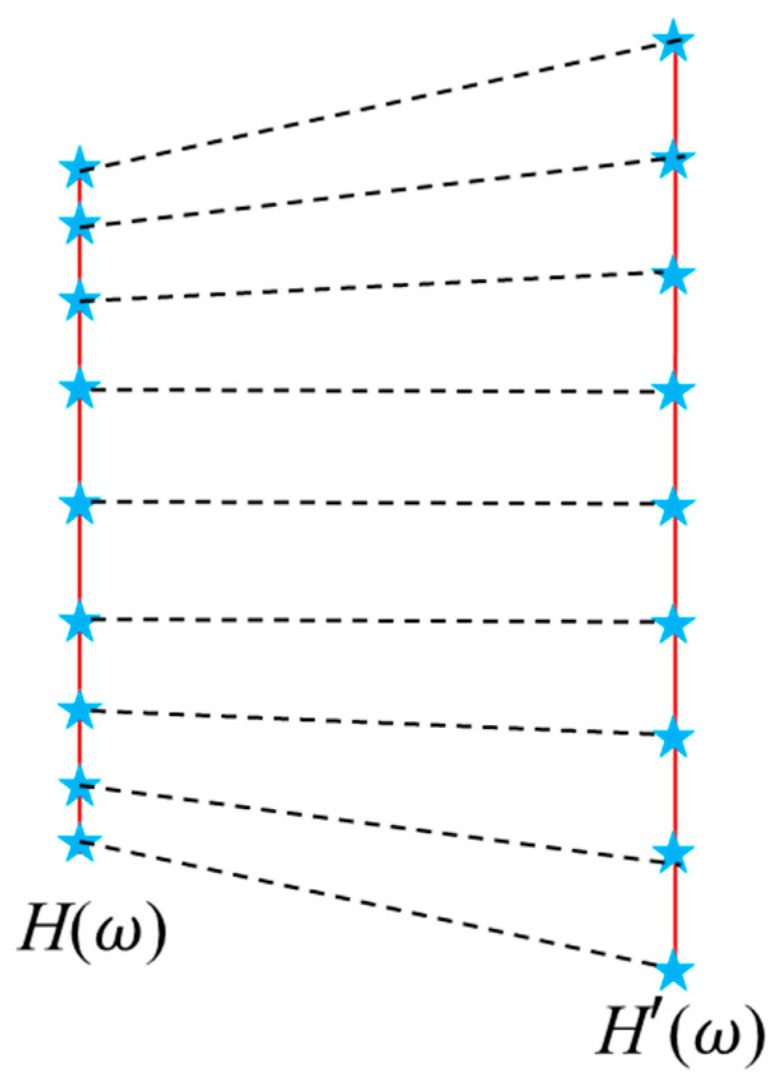
Repositioning of the stars’ image.

During star pattern recognition, star sensors need to compare the captured star patterns with navigation star catalogs. Most navigation star catalogs are based on images obtained using conventional optical systems, whereas our proposed optical system’s star imaging positions are related to *FFL*(*ω*). As shown in Figure 15, repositioning is required before star pattern recognition. The relationship between the FFL-type star sensor’s image height *H*(*ω*) and the conventional optical system’s image height *H*’ (*ω*) is one to one with the field angle. Therefore, star point image repositioning can be easily achieved.

We designed two optical systems that achieve the respective goals of enlarging the FOV and enhancing the single-star measurement accuracy. However, the *FFL*(*ω*) is not unique, and it can be customized according to specific mission requirements to achieve either or both improvements in single-star measurement accuracy and FOV. Additionally, *FFL*(*ω*) with higher-order terms can be designed to control the variation rate of measurement precision with respect to the field angle, thereby enabling the star sensor to meet more complex measurement requirements.

Conventional star sensors have the same measurement accuracy across the entire FOV, whereas the measurement accuracy of FFL-type star sensors varies with the FOV. Although the single-star measurement accuracy can theoretically be determined during system design, considering uncertainties in actual manufacturing, it is still necessary to recalibrate the measurement accuracy. Calibration of the star sensor’s measurement accuracy can be achieved using grid plates, resolution plates, and polygonal targets.

The star sensor proposed in this manuscript achieves the highest single-star measurement accuracy in the central field and lower accuracy at the edge. The distribution of stars can also affect the measurement accuracy. The best working state of the star sensor is that the attitude of the spacecraft can be determined by the stars in the center FOV. However, if stars at the edge field must be used for attitude determination, the star sensor’s optical axis can be rotated through control means. It is essential to note that this rotation is not a blind rotation without any prior data. After rotation, the relative orientation between the star sensor and the spacecraft has changed, and the precise output of the change angle of attitude is necessary. In other words, even with the rotation, the relative orientation between the star sensor and the spacecraft is known. In this way, no matter where the star is, it can be moved to the center FOV, and high-precision single-star measurement accuracy can be obtained. Considering practical applications, the rotation angle of the star sensor’s optical axis can be fixed at 3~5 predetermined values.

## 5. Conclusions

A star sensor optical system based on the FFL imaging principle has been proposed, which effectively enhances both the FOV and the single-star measurement accuracy. Based on the analysis of the imaging principle, transmissive and catadioptric optical systems are designed to improve the single-star measurement accuracy and enlarge the FOV, respectively. For the transmissive system, without compromising the single-star measurement accuracy, the FOV is expanded from 10.8° to 18°, significantly increasing the star sensor’s capture space for stars. The catadioptric system, while maintaining the same FOV, improves the system’s single-star measurement accuracy from 0.02 mrad to 0.015 mrad, achieving an enhancement of 1.35 times compared to the conventional system. This type of star sensor can simultaneously meet the requirements for star pattern recognition and attitude determination, making it of great significance for spacecraft positioning, navigation, and control.

## Figures and Tables

**Table 1:** Optical parameters of conventional star sensor and FFL-type star sensor.

Parameters	Conventional Star Sensor	FFL-Type Star Sensor
Waveband	480~650 nm	
Maximum image height	2.7 mm	
Entrance pupil of the center field	9.5 mm	
Focal length of the center field	14.3 mm	15 mm
FOV	10.8°	18°
Single-star measurement accuracy	0.35 mrad	0.33 mrad

**Table 2:** Optical parameters of conventional and FFL-type R-C systems.

Parameters	Conventional Star Sensor	FFL-Type Star Sensor
Waveband	480~650 nm	
Maximum image height	18.6 mm	
Entrance pupil	170 mm	
FOV	1.8°	
Focal length of the center field	592 mm	800 mm
Single-star measurement accuracy	0.02 mrad	0.015 mrad

**Table 3:** Tolerance of the star sensors.

Tolerance Items	Value
Radius (fringes) Decenter X/Y	2 0.015 mm
Thickness	0.02 mm
Tilt X/Y	0.02°
Aspherical surface irregularity	λ/30
Index of refraction	0.0003
Abbe number	1%

## Data Availability

Not applicable.
